# Performance-Enhancing Substance Use and Intimate Partner Violence: A Prospective Cohort Study

**DOI:** 10.1177/08862605211073097

**Published:** 2022-02-04

**Authors:** Kyle T. Ganson, Dylan B. Jackson, Alexander Testa, Jason M. Nagata

**Affiliations:** 1 Factor-Inwentash Faculty of Social Work, 7938 University of Toronto, Toronto, ON, Canada; 2 Department of Population, Family, and Reproductive Health, Johns Hopkins Bloomberg School of Public Health, Baltimore, MD, USA; 3 Department of Criminology & Criminal Justice, 12346 University of Texas at San Antonio, San Antonio, TX, USA; 4 Department of Pediatrics, 8785 University of California, San Francisco, San Francisco, CA, USA

**Keywords:** performance-enhancing substance use, anabolic steroids, steroids, creatine, intimate partner violence, adults

## Abstract

Research has shown that performance-enhancing substance (PES) use, including anabolic-androgenic steroids (AAS), is associated with interpersonal violence (e.g., fighting). This study aimed to determine whether legal PES use and AAS use are associated with intimate partner violence (IPV) involvement cross-sectionally and over seven-year follow-up in a nationally representative prospective cohort study. Data from the National Longitudinal Study of Adolescent to Adult Health (N = 12,288) were analyzed (2021). Logistic regression analyses were conducted to determine the associations between legal PES use and AAS use at Wave III (2001–2002; ages 18–26) and IPV victimization (five variables) and IPV perpetration (five variables) at Wave III and Wave IV (2008–2009; ages 24–32), adjusting for relevant demographic and confounding variables. Results from cross-sectional analyses showed that legal PES use and AAS use were associated with higher odds of both any IPV victimization and sexual IPV victimization, and both any IPV perpetration and physical IPV perpetration by pushing or shoving a partner. Results from prospective analyses showed that AAS use, but not legal PES use, was associated with higher odds of all five IPV victimization variables (any IPV victimization: adjusted odds ratio [AOR] 1.72, 95% confidence interval [CI]1.04–2.84; two forms of physical abuse: 1: AOR 2.01, 95% CI 1.15–3.50; 2: AOR 2.11, 95% CI 1.06–4.21; incurring an injury from IPV victimization: AOR 4.90, 95% CI 1.71–14.01; and sexual IPV victimization AOR 2.44, 95% CI 1.05–5.65), as well as three IPV perpetration variables (any IPV perpetration: AOR 2.11, 95% CI 105–4.23; one form of physical abuse perpetration: AOR 2.58, 95% CI 1.06–6.27; and sexual IPV perpetration: AOR 3.80, 95% CI 1.44–10.02). These results emphasize the adverse social and interpersonal risks associated with PES use. Continued research, health care, and public health prevention and intervention efforts to reduce the use of PES and occurrence of IPV are warranted.

## Introduction

Performance-enhancing substances (PES), including both legal substances (e.g., protein powders and creatine monohydrate) and illegal (e.g., anabolic-androgenic steroids [AAS]) substances are commonly used among adolescent boys and young adult men. This includes 16–35% reporting use of legal PES and 3–6% reporting AAS use, compared to 3–21% and 0.4–5% of adolescent girls and young adult women who report legal PES and AAS use, respectively (Eisenberg et al., 2012; Nagata et al., 2020b). PES use is associated with a plethora of adverse health outcomes. For example, use of legal PES is associated with severe medical events (e.g., hospital visits and disability) and death (Or et al., 2019), while AAS use is associated with neuropsychiatric effects (e.g., mood disorders, irritability, and paranoia; Kanayama et al., 2008; Pope et al., 2014) and physiological health problems (Ahlgrim & Guglin, 2009; Bispo et al., 2009; Daly et al., 2003; Nikolopoulos et al., 2011). Furthermore, both legal PES and AAS use are associated with substance use problems (Ganson et al., 2020) and dependence (Ip et al., 2010, 2011) and criminal offending (Ganson et al., 2021b). What remains less well understood is how PES use, including both legal PES and AAS, impacts social functioning, such as intimate partner relationships.

Intimate partner violence (IPV) is defined by the Centers for Disease Control and Prevention as physical or sexual violence, stalking, or psychological aggression by a current or former intimate partner (including both spouses and non-spouses; Breiding et al., 2015). IPV is common among the U.S. population, where 25% of cisgender women and 11% of cisgender men reported any contact sexual victimization, physical victimization, and/or stalking with an IPV-related effect (e.g., psychological distress, social impairment, and sexually transmitted infection; Smith et al., 2015). Additionally, sexual and gender minorities (i.e., those who do not identify as heterosexual or cisgender) often experience IPV at higher prevalence than their heterosexual and cisgender peers (Swiatlo et al., 2020; Whitfield et al., 2021). Furthermore, there are disparities of IPV involvement across racial/ethnic identities, whereby Black or African Americans, compared to White or European Americans, are at greater risk of IPV (Capaldi et al., 2012). This risk may be further exacerbated by sexual and gender minority identity status (Whitton et al., 2021). This is concerning given that IPV is associated with numerous physical, psychological, and social health outcomes, including injury, chronic pain, depression, anxiety, posttraumatic stress disorder, urologic and gynecologic conditions, among others (Bacchus et al., 2018; Miller & McCaw, 2019).

The preceding data emphasize the common occurrence of both PES use and IPV and their significant health effects. To date, it remains unknown whether PES use and IPV involvement are associated. However, current evidence suggests that PES use, particularly AAS use, is associated with aggressive tendencies and the perpetration of general interpersonal violence (e.g., physical fighting; Beaver et al., 2008; Hauger et al., 2021). One study has shown that AAS is associated with teen dating violence, including physical and sexual violence, among adolescents in Massachusetts (Ganson & Cadet, 2019). Combined, these studies indicate that IPV perpetration may also occur in relation to AAS use; however, there remain gaps in the knowledge base on PES use and IPV, specifically for legal PES and identifying longitudinal associations among a national sample of participants.

Research on AAS use, not including legal PES use, has identified multiple mechanisms that may explain the link between AAS use and IPV. That is, AAS use may result in changes in brain structure in areas key to impulse control and emotion regulation (Hauger et al., 2019b; Westlye et al., 2017), an overall decrease in executive functioning abilities (Hauger et al., 2020), impaired emotion recognition (Hauger et al., 2019a), and increased anger (Hauger et al., 2021), all of which may increase one’s propensity for violence (Gottfredson & Hirschi, 1990; Raine, 2002, 2013). Thus, it is clear that AAS use can have detrimental effects on important skills needed for social functioning. Additionally, individuals who endorse a greater drive for muscularity, which is a predictor of PES use (Murray et al., 2017; Tylka, 2011), also more commonly adhere to hegemonic norms of masculinity (Griffiths et al., 2015; Luciano, 2007; McCreary et al., 2005). Such adherence to traditional hegemonic masculinity is also associated with violence (Copenhaver et al., 2000; Vandello & Bosson, 2013) and IPV (Tarzia, 2020; Willie et al., 2018). Thus, the overlapping connections between drive for muscularity, PES use, and adherence to hegemonic masculine norms may precipitate acts of IPV. Furthermore, sexual minority individuals, as with IPV, may be more likely to use AAS given unique minority stressors on these groups (Blashill et al., 2017; Calzo et al., 2016). Such minority stressors have also been shown to be contributing factors to IPV involvement among sexual minorities (Swiatlo et al., 2020; Whitfield et al., 2021; Whitton et al., 2021). Taken together, there appears to be evidence of sociocultural influences that underpin a potential relationship between PES use and IPV that may inform future research findings and contextualize prevention and intervention efforts.

It is also possible that individuals who use AAS are more susceptible to IPV victimization. This may be due to risk behaviors that AAS users engage in, such as unsafe sexual behaviors (Ip et al., 2016) and alcohol and illicit drug use (Ip et al., 2011). These behaviors may occur in high-risk social situations where the likelihood of IPV victimization is elevated. However, to date, no known research has investigated whether AAS use is associated with IPV victimization, as well as whether legal PES use is associated with IPV involvement. To fill these gaps in the knowledge base, this study aimed to determine whether legal PES use and AAS use are cross-sectionally and prospectively associated with IPV involvement among a nationally representative cohort sample of U.S. young adults. Findings may provide valuable information for health care and public health professionals to implement effective PES use and IPV prevention and intervention strategies. We hypothesized that PES, particularly AAS, would be associated with IPV involvement among young adults.

## Methods

Prospective cohort data from the National Longitudinal Study of Adolescent to Adult Health (Add Health) were analyzed. Add Health participants were enrolled in grades 7–12 (ages 12–19) in public, private, and parochial schools in the U.S. during 1994–1995 academic year. Four follow-up interview have been conducted since then. This study included participants who completed interviews at Wave I (1994–1995; ages 11–18), Wave III (2001–2002; ages 18–26), and Wave IV (2008–2009; ages 24–32; Harris, 2013; Harris et al., 2019).

### Measures

#### Independent Variable

*Legal PES use* was assessed using self-reported responses to the question, “In the past year, have you used a legal performance-enhancing substance for athletes (such as creatine monohydrate or andro)?” *AAS use* in the past 7 years (since Wave I) was assessed using self-reported responses to the question, “Since June 1995, have you taken any of the following drugs without a doctor’s permission: steroids or anabolic steroids”. Both items were assessed at Wave III (2001–2002; ages 18–26) and response options were “yes” or “no.” These items have been used in prior research (see for examples Ganson et al., 2020, 2021a, 2021b; Nagata et al., 2020a, 2020b).

### Dependent Variable

*Intimate partner violence involvement* was assessed at Wave III (2001–2002; ages 18–26) and Wave IV (2008–2009; ages 24–32) using four items of both IPV victimization and perpetration. IPV victimization was measured based on experiencing the following within the past year: “partner pushed or shoved you, or threw something at you that could hurt”; “partner slapped, hit, or kicked you”; “you had an injury, such as a sprain, bruise, or cut because of a fight with your partner”; and “partner insisted on or made you have sexual relations with him/her when you did not want to.” Response options included categories assessing frequency of occurrence ranging from no occurrence to 20 or more occurrences. Responses were dichotomized to no occurrence and any occurrence in accordance with prior literature (Dunn et al., 2021; Reingle et al., 2012; Spivey & Nodeland, 2021; Swiatlo et al., 2020). *Any IPV victimization* was assessed using a combined dichotomous variable that measured whether a participant reported experiencing any IPV victimization involvement.

IPV perpetration was measures based on experiencing the following within the past year: “you pushed or shoved, or threw something at your partner that could hurt”; “you slapped, hit, or kicked your partner”; “your partner had an injury, such as a sprain, bruise, or cut because of a fight with you”; and “you insisted on or made your partner have sexual relations with you when they did not want to.” Response options included categories assessing frequency of occurrence ranging from no occurrence to 20 or more occurrences. Responses were dichotomized to no occurrence and any occurrence in accordance with prior literature (Dunn et al., 2021; Reingle et al., 2012; Spivey & Nodeland, 2021; Swiatlo et al., 2020). *Any IPV perpetration* was assessed using a combined dichotomous variable that measured whether a participant reported engaging in any IPV perpetration.

### Demographic and Confounding Variables

Demographic variables included self-reported biological sex (1994–1995; ages 11–18; Wave I), age (2001–2002; ages 18–26; Wave III), race/ethnicity (1994–1995; ages 11–18; Wave I), sexual orientation (2001–2002; ages 18–26; Wave III), and household income (1994–1995; ages 11–18; Wave I). Confounding variables included body mass index (BMI; kg/m^2^; 2001–2002; ages 18–26; Wave III), alcohol use (≥2 days in the past month, yes/no; 2001–2002; ages 18–26; Wave III), ever lived with someone in a marriage-like relationship (yes/no; 2001–2002; ages 18–26; Wave III), ever married or lived with partner (yes/no; 2008–2009; ages 24–32; Wave IV), low self-control based on Gottfredson and Hirschi’s (1990) six dimensions of self-control (see Perrone et al., 2004 for further variable description; 1994–1995; ages 11–18; Wave I), depression score (9-item version of the Center for Epidemiologic Studies-Depression [CES-D] scale; 2001–2002; ages 18–26; Wave III; Radloff, 1977), and childhood physical abuse and sexual abuse (never/any; 2001–2002; ages 18–26; Wave III). These variables were included based on prior research showing relationships with PES use and IPV involvement (Beaver et al., 2008; Capaldi et al., 2012; Eisenberg et al., 2012; Ganson et al., 2020; Hauger et al., 2020, 2021, Ip et al., 2010, 2011; Kanayama et al., 2008; Nagata et al., 2020b; Pope et al., 2014; Singh et al., 2014).

## Statistical Analysis

Descriptive statistics were estimated to characterize the sample overall and by legal PES use and AAS use. Logistic regression analyses were conducted cross-sectionally to determine the associations between legal PES use and AAS use at Wave III as the independent variables and IPV victimization (five variables) and IPV perpetration (five variables) at Wave III as the dependent variables. Additionally, logistic regression analyses were conducted prospectively to determine the associations between legal PES use and AAS use at Wave III as the independent variables and IPV victimization (five variables) and IPV perpetration (five variables) at Wave IV as the dependent variables. All regression analyses controlled for the demographic and confounding variables, and prospective analyses additionally controlled for any IPV involvement, including both victimization and perpetration, at Wave III. We tested for effect modification/interaction for sex and PES use and found no statistically significant effects (*p* for interaction >0.05). Therefore, we did not stratify our analyses by sex. All analyses utilized Add Health’s nationally representative sample weights and were conducted in 2021 using Stata 15.1.

## Results

The sample of 12,288 participants was demographically diverse and was comprised of 50.6% men, 31.5% racial/ethnic minorities, and a mean age of 21.8 years (standard deviation 1.8). In young adulthood (18–26 years), 2.0% of participants reported AAS use in the past 7 years, while 8.8% reported legal PES use in the past year. At seven-year follow-up (24–32 years), 11.1% reported any IPV victimization and 5.9% reported any IPV perpetration. Overall, prevalence of all IPV victimization and IPV perpetration items were descriptively higher in Wave III (18–26 years) compared to Wave IV (24–32 years). Full sample characteristics are presented in Table 1.

**Table 1.** Demographic and descriptive statistics of Add Health participants (N = 12,288).

	Overall	Legal PES Users	AAS Users
	M (SD)/%	M (SD)/%	M (SD)/%
Age, ages 18–26 years (Wave III)	21.8 (1.8)	21.7 (1.8)	21.8 (2.0)
Sex, ages 11–18 years (Wave I)			
Female	49.4%	6.7%	20.8%
Male	50.6%	93.3%	79.2%
Race/ethnicity, ages 11–18 years (Wave I)			
White	68.5%	77.1%	77.3%
Hispanic/Latino	11.9%	9.5%	11.9%
Black/African American	15.2%	9.3%	9.8%
American Indian/Native American	0.5%	0.6%	<0.0%
Asian/Pacific Islander	3.1%	2.5%	0.9%
Other race/ethnicity	0.8%	0.9	0.0%
Sexual orientation, ages 18–26 years (Wave III)			
Heterosexual	90.2%	94.5%	89.4%
Mostly heterosexual	6.9%	2.9%	6.4%
Gay, lesbian, or bisexual	3.0%	2.6%	4.2%
Household income (thousands of dollars), ages 11–18 years (Wave I)	46.3 (41.8)	53.1 (38.8)	45.5 (31.0)
Body mass index (kg/m^2^), ages 18–26 years (Wave III)	26.4 (6.4)	26.2 (5.1)	27.3 (6.2)
Alcohol use, ≥ 2 days, past 30 days, ages 18–26 years (Wave III)	47.0%	69.4%	53.3%
Ever lived with someone in a marriage-like relationship, ages 18–26 years (Wave III)	41.8%	38.7%	47.7%
Ever married or lived with partner, ages 24–32 years (Wave IV)	85.4%	84.0%	91.3%
Low self-control, ages 11–18 years (Wave I)	6.5 (3.2)	6.6 (3.2)	7.1 (3.7)
Depression score, ages 18–26 years (Wave III)	4.4 (4.0)	4.0 (3.8)	5.0 (4.2)
Childhood physical abuse, ages 18–26 years (Wave III)	28.8%	34.5%	48.6%
Childhood sexual abuse, ages 18–26 years (Wave III)	4.6%	6.0%	23.5%
IPV victimization, past year, ages 18–26 years (Wave III)			
Any IPV victimization	33.3%	33.0%	47.5%
Physical IPV victimization 1^a^	23.8%	24.9%	38.3%
Physical IPV victimization 2^b^	18.0%	23.1%	30.5%
IPV victimization injury^c^	5.3%	5.6%	12.8%
Sexual IPV victimization^d^	11.8%	13.8%	25.3%
IPV perpetration, past year, ages 18–26 years (Wave III)			
Any IPV perpetration	28.2%	26.1%	38.9%
Physical IPV perpetration 1^e^	20.4%	20.4%	34.0%
Physical IPV perpetration 2^f^	15.6%	12.0%	19.5%
IPV perpetration injury^g^	5.3%	5.6%	12.8%
Sexual IPV perpetration^h^	5.7%	7.0%	10.1%
Any IPV involvement, past year, ages 18–26 years (Wave III)	37.7%	37.1%	51.4%
IPV victimization, past year, ages 24–32 years (Wave IV)			
Any IPV victimization	11.1%	11.7%	21.4%
Physical IPV victimization 1^a^	8.4%	9.9%	18.3%
Physical IPV victimization 2^b^	4.8%	6.2%	13.2%
IPV victimization injury^c^	1.6%	1.5%	5.7%
Sexual IPV victimization^d^	3.0%	3.8%	7.6%
IPV perpetration, past year, ages 24–32 years (Wave IV)			
Any IPV perpetration	5.9%	6.9%	13.5%
Physical IPV perpetration 1^e^	4.0%	4.2%	8.9%
Physical IPV perpetration 2^f^	2.6%	1.3%	2.4%
IPV perpetration injury^g^	0.9%	0.9%	1.6%
Sexual IPV perpetration^h^	1.5%	3.4%	7.1%
Any IPV involvement, past year, ages 24–32 years (Wave IV)	12.7%	14.0%	22.7%
Anabolic-androgenic steroid use, past 7 years, ages 18–26 years (Wave III)	2.0%	13.8%	–
Legal performance-enhancing substance use, past 12-months, ages 18–26 years (Wave III)	8.8%	–	61.5%

*Note.* Analyses included preconstructed sample weighting.

IPV=Intimate partner violence; PES=Performance-enhancing substance use; AAS=Anabolic-androgenic steroids.

^a^Partner pushed or shoved you, or threw something at you that could hurt (any/none).

^b^Partner slapped, hit, or kicked you (any/none).

^c^Injury, such as a sprain, bruise, or cut, because of a fight with your partner (any/none).

^d^Partner insisted on or made you have sexual relations with him/her when you did not want to (any/none).

^e^You pushed or shoved, or threw something at your partner that could hurt (any/none).

^f^You slapped, hit, or kicked your partner (any/none).

^g^Partner had an injury, such as a sprain, bruise, or cut, because of a fight with you (any/none).

^h^You insisted on or made your partner have sexual relations with you when they did not want to (any/none).

Results from cross-sectional logistic regression analyses showed significant associations between legal PES use and AAS use and IPV victimization and perpetration. Regarding IPV victimization (Table 2, Panel A), participants who reported AAS use had higher odds of any IPV victimization (adjusted odds ratio [AOR] 1.84, 95% confidence interval [CI] 1.11–3.07), physical IPV victimization (1: AOR 1.85, 95% CI 1.08–3.15), and sexual IPV victimization (AOR 2.65, 95% CI 1.44–4.87), while adjusting for demographic and behavioral variables. Participants who reported legal PES use had higher odds of any IPV victimization (AOR 1.34, 95% CI 1.04–1.72) and sexual IPV victimization (AOR 1.55, 95% CI 1.12–2.15), while adjusting for demographic and behavioral variables.

**Table 2.** Panel A: Cross-Sectional Associations between Performance-Enhancing Substance Use and Intimate Partner Violence Victimization in Young Adulthood (18–26 Years) among Participants in Add Health.

	Any IPV Victimization	Physical IPV Victimization 1^a^	Physical IPV Victimization 2^b^	IPV Victimization Injury^c^	Sexual IPV Victimization^d^
	AOR (95% CI)	AOR (95% CI)	AOR (95% CI)	AOR (95% CI)	AOR (95% CI)
Anabolic-androgenic steroid use, past 7 years	1.84 (1.11–3.07)*	1.85 (1.08–3.15)*	1.48 (0.84–2.62)	2.14 (0.93–4.96)	2.65 (1.44–4.87)**
	AOR (95% CI)	AOR (95% CI)	AOR (95% CI)	AOR (95% CI)	AOR (95% CI)
Legal performance-enhancing substance use, past 12-months	1.34 (1.04–1.72)*	1.29 (0.99–1.70)	1.28 (0.96–1.72)	1.30 (0.79–2.14)	1.55 (1.12–2.15)**
Table 2: Panel B: Cross-sectional associations between performance-enhancing substance use and intimate partner violence perpetration in young adulthood (18–26 Years) among participants in add health					
	Any IPV Perpetration	Physical IPV Perpetration 1^e^	Physical IPV Perpetration 2^f^	IPV Perpetration Injury^g^	Sexual IPV Perpetration^h^
	AOR (95% CI)	AOR (95% CI)	AOR (95% CI)	AOR (95% CI)	AOR (95% CI)
Anabolic-androgenic steroid use, past 7 years	1.70 (1.00–2.90)*	2.26 (1.27–4.04)**	1.42 (0.65–3.11)	2.14 (0.93–4.96)	1.33 (0.58–3.06)
	AOR (95% CI)	AOR (95% CI)	AOR (95% CI)	AOR (95% CI)	AOR (95% CI)
Legal performance-enhancing substance use, past 12-months	1.36 (1.02–1.81)*	1.54 (1.15–2.06)**	1.53 (1.01–2.33)*	1.30 (0.79–2.14)	1.18 (0.79–1.75)

*Note.* Analyses included preconstructed sample weighting.

**Boldface** indicates statistical significance (*p* < 0.05); * *p* < 0.05 ** *p* < 0.01.

IPV=Intimate partner violence; AOR=Adjusted odds ratio; CI=Confidence interval.

^a^Partner pushed or shoved you, or threw something at you that could hurt (any/none).

^b^Partner slapped, hit, or kicked you (any/none).

^c^Injury, such as a sprain, bruise, or cut, because of a fight with your partner (any/none).

^d^Partner insisted on or made you have sexual relations with him/her when you did not want to (any/none).

^e^You pushed or shoved, or threw something at your partner that could hurt (any/none).

^f^You slapped, hit, or kicked your partner (any/none).

^g^Partner had an injury, such as a sprain, bruise, or cut, because of a fight with you (any/none).

^h^You insisted on or made your partner have sexual relations with you when they did not want to (any/none).

All analyses adjusted for age (W III), sex (W I), race/ethnicity (W I), sexual orientation (W III), household income (W I), body mass index (W III), alcohol use (W III), relationship status (W III), low self-control (W I), depression score (W III), childhood physical abuse (W III), childhood sexual abuse (W III).

Regarding IPV perpetration (Table 2, Panel B), participants who reported AAS use had higher odds of any IPV perpetration (AOR 1.70, 95% CI 1.00–2.90) and physical IPV perpetration (1: AOR 2.26, 95% CI 1.27–4.04), while adjusting for demographic and behavioral variables. Participants who reported legal PES use had higher odds of any IPV perpetration (AOR 1.36, 95% CI 1.02–1.81) and both forms of physical violence perpetration (1: AOR 1.54, 95% CI 1.16–2.06; 2: AOR 1.53, 95% CI 1.01–2.33), while adjusting for demographic and behavioral variables.

Results from prospective logistic regression analyses showed significant associations between AAS use in young adulthood and both IPV victimization and IPV perpetration at seven-year follow-up. Regarding IPV victimization (Table 3, Panel A), participants who reported AAS use in young adulthood had higher odds of any IPV victimization (AOR 1.72, 95% CI 1.04–2.84), both physical IPV victimization items (1: AOR 2.01, 95% CI 1.15–3.50; 2: AOR 2.11, 95% CI 1.06–4.21), IPV victimization injury (AOR 4.90, 95% CI 1.71–14.01), and sexual IPV victimization (AOR 2.44, 95% CI 1.05–5.65) at seven-year follow-up, while adjusting for demographic and behavioral variables.

**Table 3.** Panel A: Prospective Associations between Performance-Enhancing Substance Use in Young Adulthood (18–26 Years) and Intimate Partner Violence Victimization at Seven-Year Follow-Up (24–32 Years) among Participants in Add Health.

	Any IPV Victimization	Physical IPV Victimization 1^a^	Physical IPV Victimization 2^b^	IPV Victimization Injury^c^	Sexual IPV Victimization^d^
	AOR (95% CI)	AOR (95% CI)	AOR (95% CI)	AOR (95% CI)	AOR (95% CI)
Anabolic-androgenic steroid use, past 7 years	1.72 (1.04–2.84)*	2.01 (1.15–3.50)*	2.11 (1.06–4.21)*	4.90 (1.71–14.01)**	2.44 (1.05–5.65)*
	AOR (95% CI)	AOR (95% CI)	AOR (95% CI)	AOR (95% CI)	AOR (95% CI)
Legal performance-enhancing substance use, past 12-months	0.94 (0.69–1.26)	1.04 (0.78–1.40)	0.90 (0.60–1.34)	1.53 (0.65–3.62)	1.20 (0.69–2.10)
Table 3: Panel B: Prospective associations between performance-enhancing substance use in young adulthood (18–26 Years) and intimate partner violence perpetration at seven-year follow-up (24–32 Years) among participants in add health					
	Any IPV Perpetration	Physical IPV Perpetration 1^e^	Physical IPV Perpetration 2^f^	IPV Perpetration Injury^g^	Sexual IPV Perpetration^h^
	AOR (95% CI)	AOR (95% CI)	AOR (95% CI)	AOR (95% CI)	AOR (95% CI)
Anabolic-androgenic steroid use, past 7 years	2.11 (1.05–4.23)*	2.58 (1.06–6.27)*	0.64 (0.21–1.94)	1.60 (0.20–12.69)	3.80 (1.44–10.02)**
	AOR (95% CI)	AOR (95% CI)	AOR (95% CI)	AOR (95% CI)	AOR (95% CI)
Legal performance-enhancing substance use, past 12-months	1.26 (0.84–1.90)	1.52 (0.86–2.71)	0.94 (0.40–2.22)	1.55 (0.54–4.46)	1.46 (0.85–2.50)

*Note.* Analyses included preconstructed sample weighting.

**Boldface** indicates statistical significance (*p* < 0.05); * *p* < 0.05 ** *p* < 0.01.

IPV=Intimate partner violence; AOR=Adjusted odds ratio; CI=Confidence interval.

^a^Partner pushed or shoved you, or threw something at you that could hurt (any/none).

^b^Partner slapped, hit or kicked you (any/none).

^c^Injury, such as a sprain, bruise, or cut, because of a fight with your partner (any/none).

^d^Partner insisted on or made you have sexual relations with him/her when you did not want to (any/none).

^e^You pushed or shoved, or threw something at your partner that could hurt (any/none).

^f^You slapped, hit or kicked your partner (any/none).

^g^Partner had an injury, such as a sprain, bruise, or cut, because of a fight with you (any/none).

^h^You insisted on or made your partner have sexual relations with you when they did not want to (any/none).

All analyses adjusted for age (W III), sex (W I), race/ethnicity (W I), sexual orientation (W III), household income (W I), body mass index (W III), alcohol use (W III), relationship status (W IV), low self-control (W I), depression score (W III), childhood physical abuse (W III), childhood sexual abuse (W III), and any IPV involvement (W III).

Regarding IPV perpetration (Table 3, Panel B), participants who reported AAS use in young adulthood had higher odds of any IPV perpetration (AOR 2.11, 95% CI 1.06–4.23), physical IPV perpetration (1: AOR 2.58, 95% CI 1.06–6.27), and sexual IPV perpetration (AOR 3.80, 95% CI 1.44–10.02) at seven-year follow-up, while adjusting for demographic and behavioral variables. There were no statistically significant associations between legal PES use in young adulthood and IPV victimization nor IPV perpetration at seven-year follow-up.

## Discussion

The aim of this study was to determine the cross-sectional and prospective associations between PES use and IPV victimization and IPV perpetration among a nationally representative cohort sample of U.S. young adults. Results showed patterns of association between legal PES use and AAS use and IPV involvement. Specifically, in cross-sectional analyses, both legal PES use and AAS use were associated with any IPV victimization and sexual IPV victimization, while AAS use was associated with physical IPV victimization by way of being pushed or shoved by an intimate partner. Similarly, both legal PES use and AAS use were associated with any IPV perpetration and physical IPV perpetration by way of having pushed or shoved an intimate partner, while legal PES use was also associated with physical IPV perpetration by way of having slapped or hit an intimate partner. Interestingly, however, legal PES use was no longer associated with IPV victimization nor perpetration in prospective analyses. However, AAS use was prospectively associated with all four forms of IPV victimization, as well as any IPV perpetration, physical IPV perpetration by way of having pushed or shoved an intimate partner, and sexual IPV perpetration.

Taken together, these are novel findings and expand prior research in several key ways. First, cross-sectional associations between legal PES use and both IPV victimization and perpetration add to the growing literature on the adverse correlates of their use (Ganson et al., 2020, 2021b; Nagata et al., 2020a). Second, associations between AAS use and both IPV victimization and perpetration expand prior research that has shown that AAS use is associated with interpersonal violence perpetration, such as physical fighting and aggression, and victimization (e.g., sexual abuse; Beaver et al., 2008; Ganson et al., 2021b; Ganson & Cadet, 2019; Gruber & Pope, 1999; Hauger et al., 2021; Ip et al., 2010, 2011). Third, the association between AAS use and both IPV victimization and perpetration is particularly salient as these findings were observed both cross-sectionally and longitudinally, while adjusting for potential confounding factors including prior IPV involvement, childhood abuse, and psychosocial functioning (i.e., low self-control and depression). The longitudinal findings are additionally robust given that the AAS use item assessed a seven-year retrospective period, indicating the potential longevity of the impacts of AAS use. To date, little research has explored the association between AAS use and IPV, particularly IPV perpetration, with one study showing positive cross-sectional associations between AAS use and teen dating violence among a sample of high school students (Ganson & Cadet, 2019). Fourth, the sample comprised of a diverse and nationally representative sample of U.S. adults, underscoring the application of the findings to multiple identity groups (i.e., sexual, gender, and racial/ethnic minorities), that are uniquely susceptible to IPV and PES use (Blashill et al., 2017; Capaldi et al., 2012; Nagata et al., 2020b; Swiatlo et al., 2020; Whitfield et al., 2021). Overall, the findings from this study underscore the need for more research on the adverse health and social effects of PES use, as well as prevention and intervention efforts to mitigate the effects of use.

The differences between the cross-sectional and prospective findings from this study warrant further contextualization. While the association between legal PES use and IPV involvement is significant cross-sectionally, this diminishes over time. Conversely the association between AAS use and IPV involvement is made more robust over time. It may be that the physical effects of legal PES use are not as long lasting as the physical effects of AAS use. For example, specifically regarding IPV perpetration, research has shown that AAS use can have neurological effects, whereby several key areas and systems of the brain are altered. For example, AAS use may affect the serotonergic system and dopaminergic pathways, which may influence aggressive behaviors (Pope et al., 2014). Other neurological effects include reduced emotion regulation, impulse control (Hauger et al., 2019b; Westlye et al., 2017), executive functioning (Hauger et al., 2020), and emotion recognition, which can increase anger (Hauger et al., 2019a). Thus, it is likely that the neurological effects of AAS use may increase risk for IPV perpetration due to a deficit in key skills needed for effective interpersonal relationships. The cross-sectional relationship between legal PES use and IPV perpetration therefore may be better explained by social factors. For example, legal PES use, particularly among men, is likely influenced by desires to increase muscle-mass and strength (Murray et al., 2017; Tylka, 2011), and there are overlaps between muscularity and adherence to traditional hegemonic norms of masculinity, such as aggression, dominance, and strength (Gattario et al., 2015; Luciano, 2007). In turn, greater adherence to these norms may increase engagement in IPV perpetration (Tarzia, 2020; Willie et al., 2018). Ultimately, it may be that these social factors are diminished by other social factors (e.g., positive intimate relationships and reduced or ceased use of legal PES). Future research using a shorter follow-up period (i.e., less than 7 years) may better describe whether legal PES use is prospectively associated with IPV perpetration.

The associations between PES use and IPV victimization are novel and prior research on correlates of PES use may provide greater context to these findings. Regarding AAS use, given the illicit and risky nature of AAS, it is likely that individuals who use AAS are involved in social relationships and social situations that may be unsafe or uncertain (Pope et al., 2017); that is, where the likelihood of IPV occurring is high. For example, individuals who use AAS are more likely to engage in high-risk sexual behavior (Ip et al., 2016) and report alcohol and illicit drug use (Ip et al., 2011). Theoretically, lifestyle and routine activities theories that suggest persons who engage in riskier behaviors are at an elevated risk for victimization (Cohen & Felson, 1979; Hindelang et al., 1978). Lastly, research has shown that AAS exposure can elicit a decrease in fear reactions (e.g., escape and freezing responses) in stressful situations among rats (Johansson et al., 2000; Steensland et al., 2005). This may indicate that those who used AAS may have a decreased ability to protect themselves or avoid IPV victimization. Regarding the cross-sectional association between legal PES use and IPV victimization, the use of legal PES as a mechanism to increase muscle-mass and strength, which in the context of IPV victimization may be intended to protect again further interpersonal violence. For men specifically, being a part of a violent relationship may be a threat to their masculinity, as posited by precarious manhood (Vandello et al., 2008). Men whose masculinity is threatened via IPV victimization may seek to increase their muscularity and strength to protect themselves psychologically. This has been exemplified in research showing that men whose masculinity is threatened often exaggerate their strength (Frederick et al., 2017). Prior research has also shown that women may use AAS for the purpose of protecting against sexual assault (Gruber & Pope, 1999), which may be extended to the use of legal PES. Ultimately, future research is needed to better explain and contextualize the findings related to PES use and IPV victimization.

The findings from this study have important implications for health care and public health professionals. Health care professionals should be aware of the relationship between PES use and IPV involvement. Specifically, given the ease of access and widespread use of legal PES, it may be particularly important that health care professionals are screening for IPV involvement among those who report legal PES use. Relatedly, identifying AAS use and providing education on the potential physiological and social harms of use, including IPV involvement, is needed, as well as any medical treatment to mitigate the physiological effects of AAS use. Public health awareness and prevention programming should be used to reduce the use of PES (Elliott & Goldberg, 1996; Goldberg et al., 1991) and IPV (Niolon et al., 2017) and emphasize the potential detrimental interpersonal effects of PES use.

There are several limitations that should be noted of this study. First, all of the variables are based on retrospective self-report, which may increase the risk of reporting and recall bias. The risk of reporting bias may be particularly salient for reports of IPV perpetration. Second, there is the potential for unmeasured confounders that may influence the relationships between the key variables under study. However, we did adjust for several variables that are likely to influence the association between PES use and IPV involvement. This includes prior IPV involvement, alcohol use, childhood abuse, low self-control, depression, as well as demographic identifiers that have been shown to increase risk of PES use and IPV, such as sex and sexual orientation. Third, while Add Health has collected data on couples, our analyses were limited to the entire Add Health sample given the rarity of some of our measures (e.g., AAS use). This aligns with prior research on IPV (Dunn et al., 2021; Reingle et al., 2012; Spivey & Nodeland, 2021; Swiatlo et al., 2020). Similarly, we were limited in our analyses to investigating associations among the overall sample. Given the higher prevalence of AAS use and IPV among sexual, gender, and racial/ethnic diverse individuals, future research is warranted to explore these associations among diverse samples. Furthermore, we dichotomized the IPV involvement items in accordance with prior literature (Dunn et al., 2021; Reingle et al., 2012; Spivey & Nodeland, 2021; Swiatlo et al., 2020). However, this may have reduced the detail of information. Finally, the legal PES use item combined multiple substances (e.g., creatine and androstenedione), which reduced our ability to identify nuances of association with IPV involvement. Additionally, while androstenedione was considered a legal substance in the U.S. at the time of data collection (2001–2002), this substance was banned in 2004.

## Conclusion

The results from this study are among the first to show associations between PES use and IPV involvement. Cross-sectional findings showed the association between both legal PES use and AAS and IPV victimization and perpetration, while prospective associations underscored that AAS use was associated with both IPV victimization and perpetration across a seven-year period. These results expand on prior research emphasizing the relationship between PES use, particularly AAS use, and aggression, anger, and general interpersonal violence. Future research is needed to extrapolate and describe the specific frequency, dose, and duration of PES use that may further increase risk of IPV involvement.
